# Long-term study on survival and development of successive generations of *Mytilus galloprovincialis* cryopreserved larvae

**DOI:** 10.1038/s41598-022-17935-0

**Published:** 2022-08-10

**Authors:** P. Heres, J. Troncoso, E. Paredes

**Affiliations:** Laboratorio de Ecoloxía Costeira (ECOCOST), Departamento de Ecoloxía e Bioloxía Animal, Centro de Investigación Mariña, Universidade de Vigo, Vigo, Spain

**Keywords:** Animal biotechnology, Biological techniques, Biotechnology, Cell biology, Ecology, Biodiversity, Conservation biology

## Abstract

Shellfish aquaculture needs the development of new tools for the improvement of good practices avoiding the reliance on natural spat collection to increase production efficiently. The aim of this work was to improve the cryopreservation protocol for *Mytilus galloprovincialis* larvae described in Paredes et al. (in: Wolkers, Oldenhof (eds) Cryopreservation and freeze-drying protocol, methods in molecular biology, Humana Press, 2021, pp 2180, 10.1007/978-1-0716-0783-1_18). Moreover, the capability of producing adult mussels from cryopreserved 72 h-old D-larvae and potential long-term effects of cryopreservation through progenies were evaluated. The selection of 72-h old D-larvae for cryopreservation yielded 75% of recovery, higher than 50% from trochophores. The best combination was 10% Ethylene–Glycol + 0.4 M Trehalose in Filtered Sea Water (FSW) with cooling at − 1 °C/min and a water bath at 35 °C for thawing. Sucrose (SUC) solutions did not improve larval recovery (p > 0.05). At settlement, 5.26% of cryopreserved F1 larvae survived and over 70% settled. F2 cryopreservation produced 0.15% survival of spat and settlement varied from 35 to 50%. The delay of shell size showed on cryopreserved larvae declined throughout larval rearing without significant differences with controls from settlement point (p > 0.05). Long-term experiments showed that it is possible to obtain adult mussels from cryopreserved larvae and this tool does not compromise the quality of following progenies, neither for cryopreservation nor post-thawing development of them.

## Introduction

Shellfish aquaculture is mostly reliant to catches of wild seed for hatchery spat production, normally concentered in areas with high stocking biomass^1^. For example, this is the case of the culture of the Mediterranean mussel (*Mytilus galloprovincialis*), one of the most farmed mollusks worldwide. The adults release gametes to the natural environment, where external fertilization occurs. The fertilized eggs develop into ciliated trochophore larvae (18–20 h post-fertilization at 18 ± 1 °C). After 24 h, trochophores metamorphose into D-larvae, with more tissue complexity, including a protective shell with a D shape, called prodisoconch I. After 20–24 days post-fertilization, larvae reach the oldest stage, called pediveliger, and develop an eye spot and a foot organ used as indicators of their readiness for settlement. A few days later, larvae metamorphose into juveniles, when there is a reorganization of internal structures^2^. For hatchery spat production, the natural seed is collected from hard substrates and settled on culture ropes to allow its growth until the juveniles reach commercial sizes. However, this traditional activity has now encountered several problems, some of which are related to changes in the natural recruitment (e.g., seasonal patterns leading short but very intense harvesting periods. Moreover, shellfish recruitment suffers variations through the year due to several factors, for example: environmental fluctuations, demographic growth of pathogens, competition or predation, maritime transport, pollution, and the limitations imposed by seasonal spawning^3,4^. In addition, shellfish biomass does not vary only through the year, but also across locations and coastal areas; for example, it is well known that natural spat production is limited to the Atlantic coast with often variable production^5^. Variable production is also affected by incompatibility of coastal uses to wild spat production, for example high density of maritime transport, agriculture (irrigation) activities close to coastal areas, residential or industrial facilities, and other harvesting activities of related shellfisheries. Therefore, regulatory protection at local, national, and European levels has increased significantly in an attempt to preserve natural habitats and wild flora and fauna affecting the shellfish industry^6^. Another important factor is adult fitness: the adult population must be under good conditions to ensure the normal development of following generations. Along these lines, mass mortality events (close to 100%) have impacted European blue mussel production since 2014^7,8^. The main cause seems to be genomic abnormalities related to stock origin but could be also associated with environmental contaminations or a sign of disseminated neoplasia disorder^9,10^. These events are not exclusive to mussel species and also affect Pectinids^11–13^ and cockles^14^.

These barriers threaten the aquaculture sector nowadays, and climate change should be also considered for further conservation of natural mussel populations, whose potential effect is not clear in the near future. These obstacles do not only affect the aquaculture sector, but also research. This species is used extensively in ecotoxicological bioassays and monitoring programs because it is well known as a biological indicator due to its sensitivity to environmental stressors^15–17^.

The uncertain status of shellfish recruitment in the foreseeable future requires the development of methodologies to ensure the sustainability of competent seed that can be collected at the time of need. Research has been focused on the development of methodologies capable of producing spat inland in an efficient way, given the possibility of maintaining a hatchery with optimal conditions and, simultaneously, diminishing the time needed to achieve commercial size. Genetic studies, such as selective breeding programs, have been considered in order to obtain progenies more tolerant to farming, able to develop faster than others and reach the biggest sizes with minimal economic costs^18–21^. Cryopreservation has become a well-known technique that is useful for the efficient implementation of genetic approaches in this sector^1,22^. Cryopreserved samples could be able available as needed, providing flexibility for crosses on demand, avoiding the seasonal limitations^23^. Larval cryopreservation is an effective method to store both parental genetic information^24–27^.

There are several works focused on cryopreservation of mussel species. Sperm cryopreservation protocols have been developed^28–30^, although there is a lack of standardization among research centers and countries worldwide. In addition, more research is needed in order to minimize the amount of sperm required to obtain maximal fertilization rates post-thawing. Oocytes are the most difficult cell type to cryopreserve due to their sensitivity to Cryoprotecting Agents (CPAs), maybe related to the high CPA affinity to lipids that could potentially destabilize cell membranes^31–33^.

In marine cryopreservation, most of the published works are normally interested in the short-term effects of cryopreservation. Recently, several studies have focused on potential long-term effects of mollusk larval cryopreservation to develop reliable methodologies to enhance the aquaculture production by the culture of cryopreserved larvae until the settlement stage^1,34–36^.

The present work aimed to optimize a cryopreservation protocol for blue mussel larva, based on the model protocol described in Paredes et al.^37^. By examining: different larval stages, cooling and thawing rates, as well as different Cryoprotecting Agent (CPA) combinations and variations in the CPA removal step in short-term experiments. A long-term experiment tested the capacity of the optimized protocol to produce mussel spat from cryopreserved mussel larvae, and for the first time, the development of the resulting mussel juveniles was monitored when cultured on traditional rafts in the natural environment. A final long-term experiment was carried with the subsequent (F2) generation that was cryopreserved using the same optimized cryopreservation protocol.

## Results

### Short-term experiments

#### Potential toxic and cryoprotection effects of different CPA combinations

**Figure 1:**
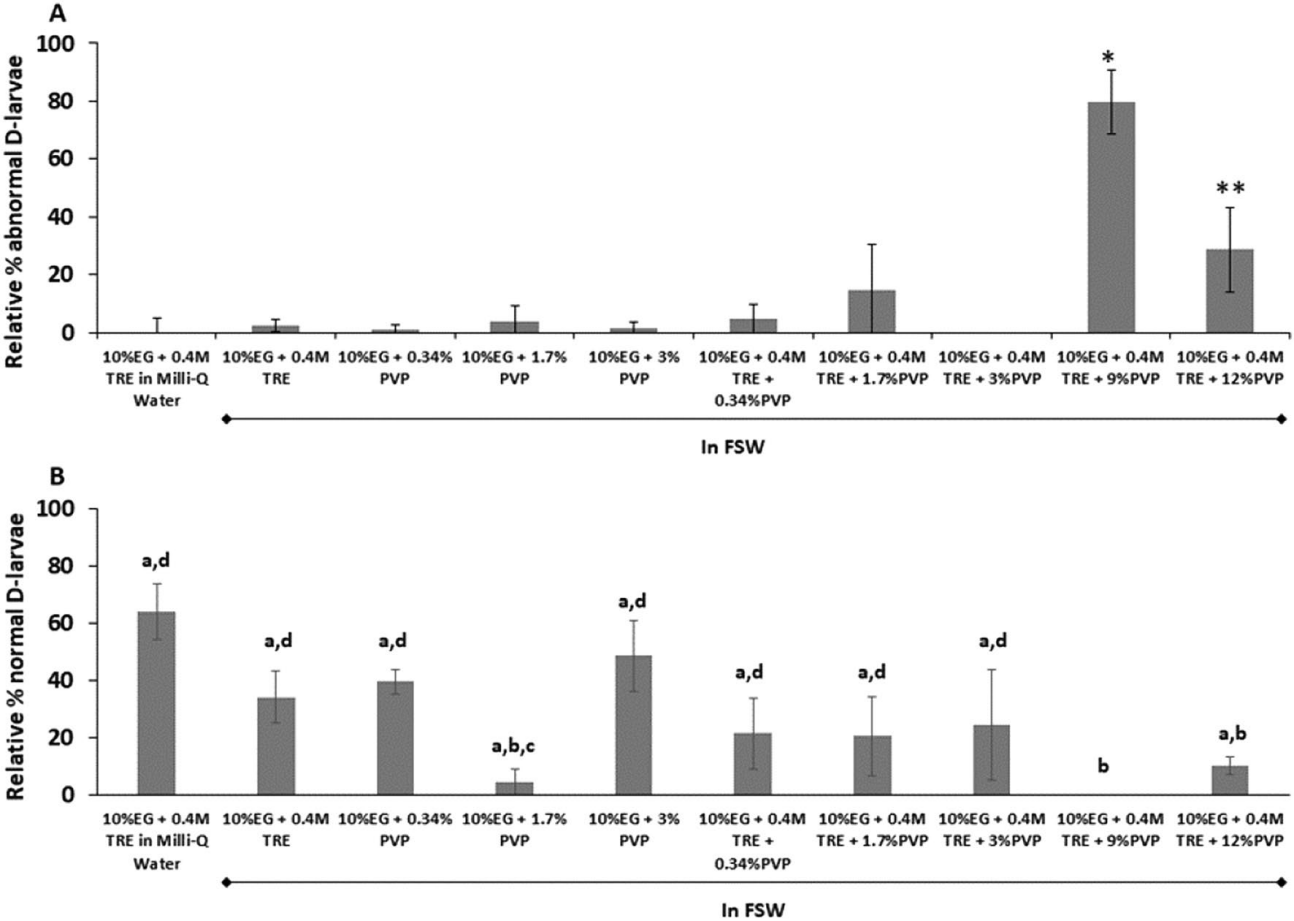
Cryopreservation experiments with *Mytilus galloprovincialis* trochophore larvae to test the effect of different CPA combinations. (**A**) Relative percentage of abnormal D-larvae compared to the control group developed 24 h from trochophores exposed for 15 min to different cryoprotecting combinations of Ethylene–Glycol (EG), trehalose (TRE) and Polyvinylpyrrolidinone (PVP) in Filtered Sea Water (FSW) or Milli-Q Water (n = 100, 3 replicates per treatment) Mean ± Standard Deviation (SD) (% of abnormal larvae in controls: 20 ± 4.58). (**B**) Percentage of trochophores developing to normal D-larvae following cryopreservation. Cryopreservation success was assessed as the average percentage of normal D-larvae after 24 h post-cryopreservation in comparison to the control group (n = 100, 3 replicates per treatment) Mean ± Standard Deviation (SD). Asterisks show significant differences between control group and treatments. Different letters show significant differences among treatments (p < 0.05) and in all cases lower % of normal larvae than controls (80 ± 4.04%).

**Figure 2:**
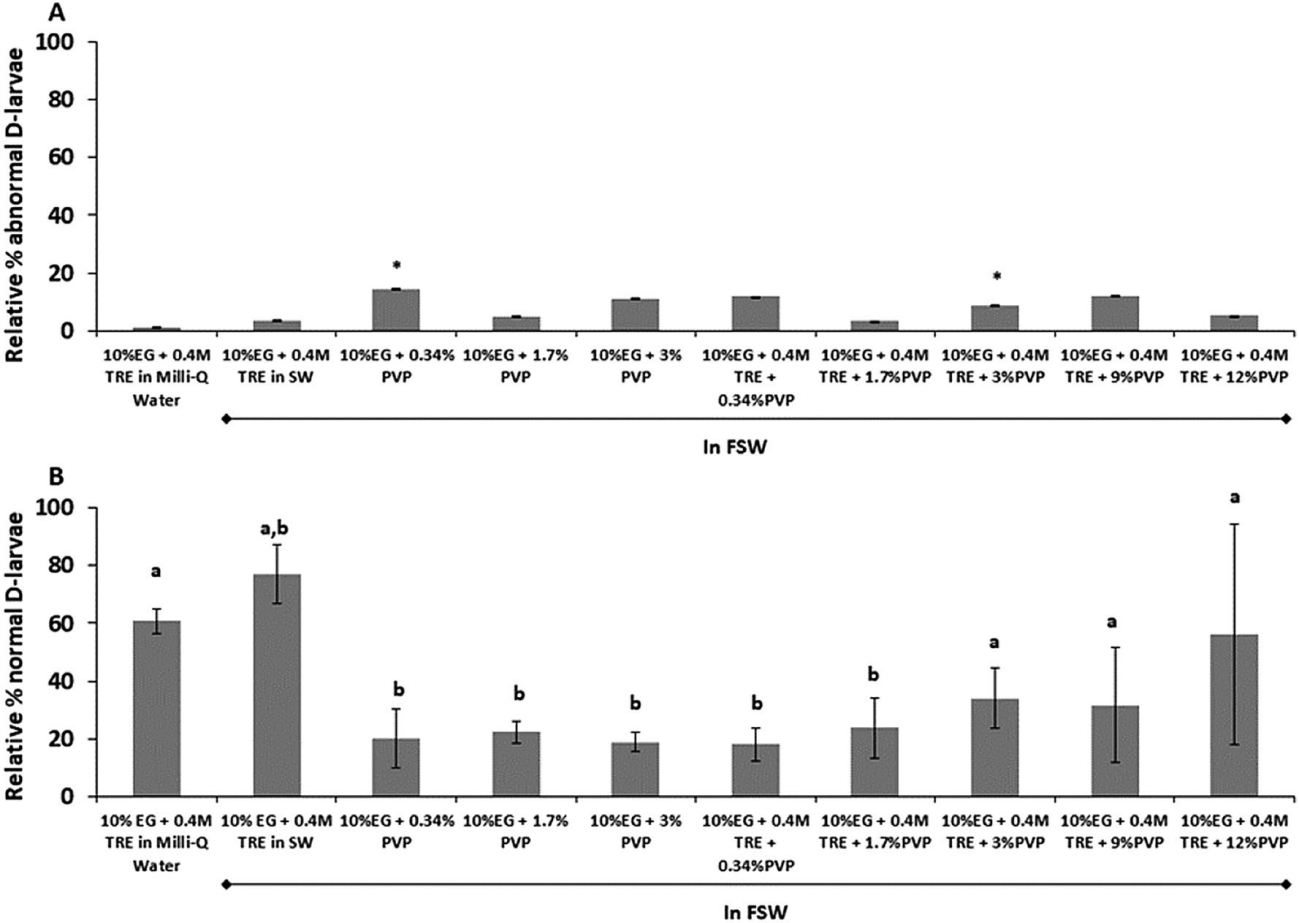
Cryopreservation experiments with *Mytilus galloprovincialis* 72 h-old D-larvae to test the effect of different CPA combinations. (**A**) Relative percentage of abnormal D-larvae compared to the control group developed 48 h from 72 h-old D-larvae exposed for 60 min to different cryoprotecting combinations of Ethylene–Glycol (EG), trehalose (TRE) and Polyvinylpyrrolidone (PVP) in Filtered Sea Water (FSW) or Milli-Q Water in 72 h-old D-larvae (% of abnormal larvae in controls: 15 ± 9.29). (**B**) Percentage of 72 h-old D-larvae developing to normal D-larvae following cryopreservation. Cryopreservation success was assessed as the average percentage of normal D-larvae after 48 h post cryopreservation in comparison to control group (Mean ± Standard Deviation (SD), n = 100, 3 replicates per treatment). Asterisks or letters show significant differences between control group and treatments. Different letters or asterisks show significant differences with p < 0.05 and in all cases lower % of normal larvae than controls (93 ± 6.33%).

Focusing on toxicity bioassays (Figs. 1A, 2A), although there were certain CPA combinations that yielded significant abnormality percentages compared to controls, in general the CPA combinations did not yield any significant toxic effect. The use of Milli-Q Water instead of FSW did not enhance normal larval development after CPA exposure, neither did the addition of PVP at the concentrations tested, even in combination with trehalose (TRE) (p > 0.05). In fact, the highest concentrations of PVP used in this experiment (9 and 12%) yielded significant abnormal development on exposed trochophores (Fig. 1A) (p < 0.05). The abnormality rates found in D-larvae developed from exposed 72 h-old D-stage did not show any dose–response relationship and only the CPA solutions containing 10% EG + 0.34% PVP with or without TRE showed significant differences with controls (Fig. 2A) (p < 0.05).

On the other hand, cryopreservation success (Figs. 1B, 2B) was significantly lower than control recovery after post-thawing incubation (p < 0.05). In general, those trials cryopreserved without PVP yielded significantly higher survival rates than the rest of the treatments regardless of the larval stage selected for cryopreservation (p < 0.05). The best survival rate from trochophore cryopreservation was 64 ± 9.66%, using 10% EG + 0.4 M TRE in Milli-Q Water (Fig. 1B). In the case of D-stage larvae, cryopreservation with 10% EG + 0.4 M TRE in FSW or Milli-Q water and 10% EG + 0.4 M TRE + 12% PVP did not show significant differences with survival of control larvae (p > 0.05). The treatment 10% EG + 0.4 M TRE in FSW had the highest survival percentage, 77 ± 4.31% (Fig. 2B).

#### Effect of different cooling and thawing rates

**Figure 3:**
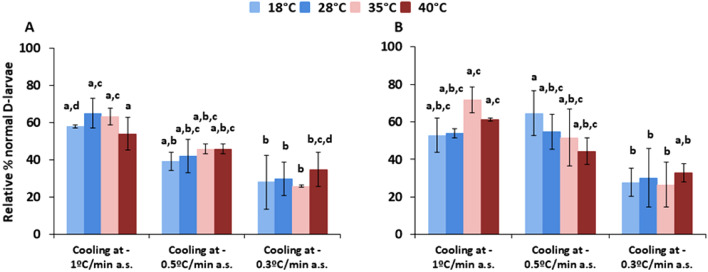
Relative percentage of *M. galloprovincialis* normal D-larvae compared to control group developed from cryopreserved trochophores (**A**) and 72 h-old D-larvae (**B**) using different cooling rates after the seeding (a.s.) check (− 1 °C/min, − 0.5 °C/min and − 0.3 °C/min) tested in combination with increasing thawing baths at 18 °C, 28 °C, 35 °C and 40 °C for melting the ice. Results are expressed as Mean ± Standard Deviation (SD), n = 100, 3 replicates per treatment. Letters show significant differences between treatments (p < 0.05).

There was a significant decrease in survival rates of cryopreserved samples compared to control groups (80%) (p < 0.05). The cooling and thawing rate combination with the highest post-thaw survival percentage was cooling at − 1 °C/min after seeding (a.s.) and the use of a water bath at 35 °C for warming of the cryopreserved straws (Fig. 3), regardless the development stage selected. In this experiment, 63 ± 4.48% of normal D-larvae were obtained from trochophore cryopreservation and 71 ± 6.88% after cryopreservation using 72 h-old D-larvae. Slower cooling rates enhanced significantly lethal injuries and larval abnormalities. Meanwhile, the highest and the lowest thawing rates did not improve cryopreservation outcome (p > 0.05).

#### Effect of SUC solutions on post-thaw D-larvae at the CPA dilution step

**Figure 4:**
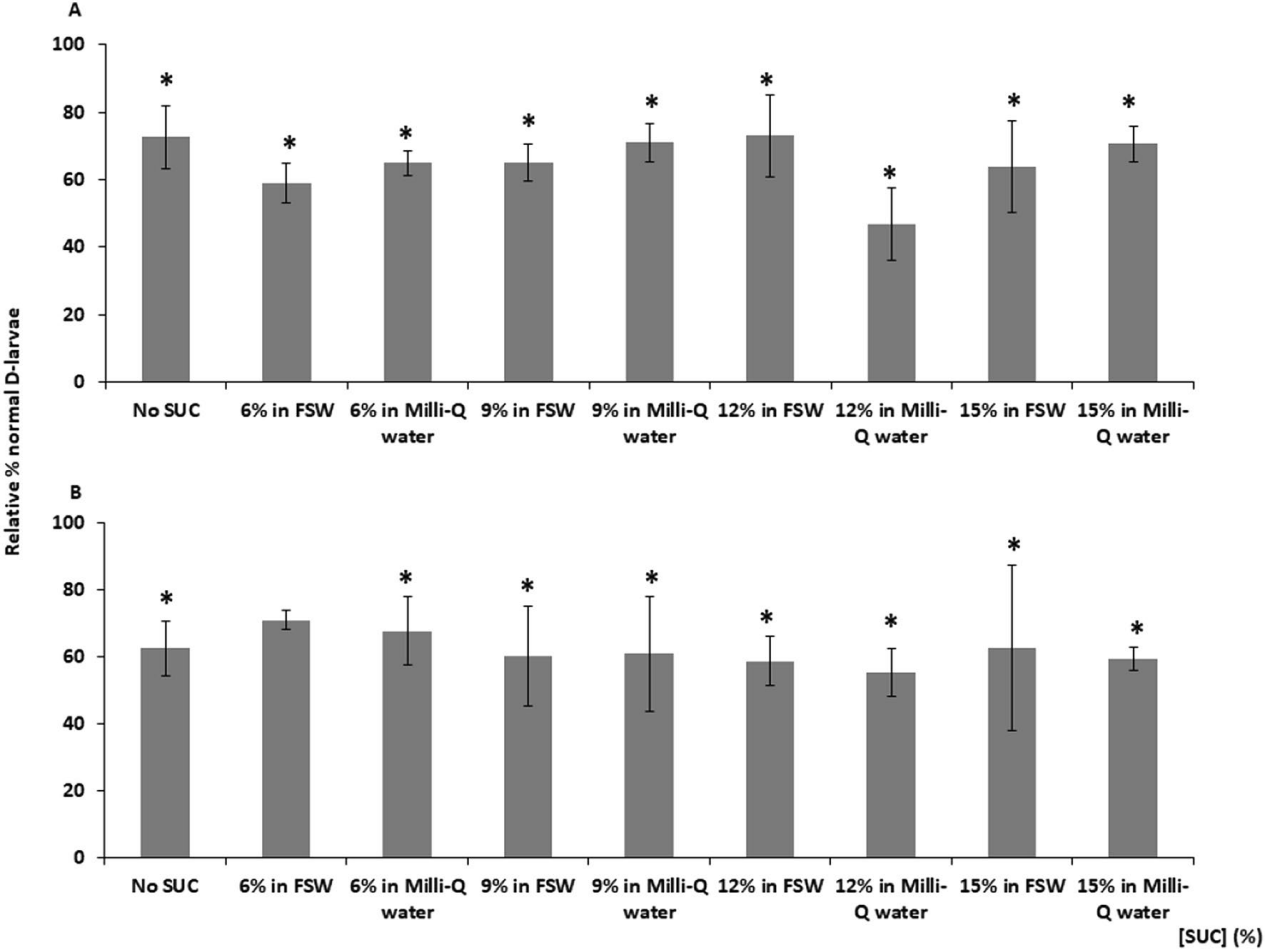
Relative percentage of *M. galloprovincialis* normal D-larvae compared to control group developed from cryopreserved trochophores (**A**), 72 h-old D-larvae (**B**), diluted with increasing sucrose (SUC) solutions in Milli-Q water or Filtered Sea Water (FSW) for CPA removal once samples were thawed. Results are expressed as Mean ± Standard Deviation (SD), n = 100, 3 replicates per treatment. Asterisk shows significant differences with control group (p < 0.05).

In agreement with prior experiments, there was a significant decrease in survival rates of post-thaw cryopreserved larvae compared to controls, except when the cryopreserved 72 h-old D-larvae were added to the solution 6% SUC in FSW for CPA dilution. However, the CPA combinations did not significantly enhance the cryopreservation success and there was not any significant effect when using SUC solutions in Milli-Q water or FSW for dilution of CPA at both larval stages (Fig. 4).

The best post-thaw performance across short-term experiments was obtained when 72 h-old D-larvae were cryopreserved in 10% EG + 0.4 M TRE in FSW with 60 min for equilibration; followed by holding at 4 °C for 2 min, then cooling at − 1 °C/min to − 12 °C, holding for 2 min for seeding check, then cooling at − 1 °C/min to − 35 °C, and finally plunging into liquid nitrogen. The best thawing protocol was using a water bath at 35 °C, and once the ice was melted, the content of the straws was diluted in FSW at 1:1. The optimized protocol was selected to further long-term experiments.

### Long-term experiments

#### Long-term experiment from cryopreserved F1 72 h-old D-larvae

**Figure 5:**
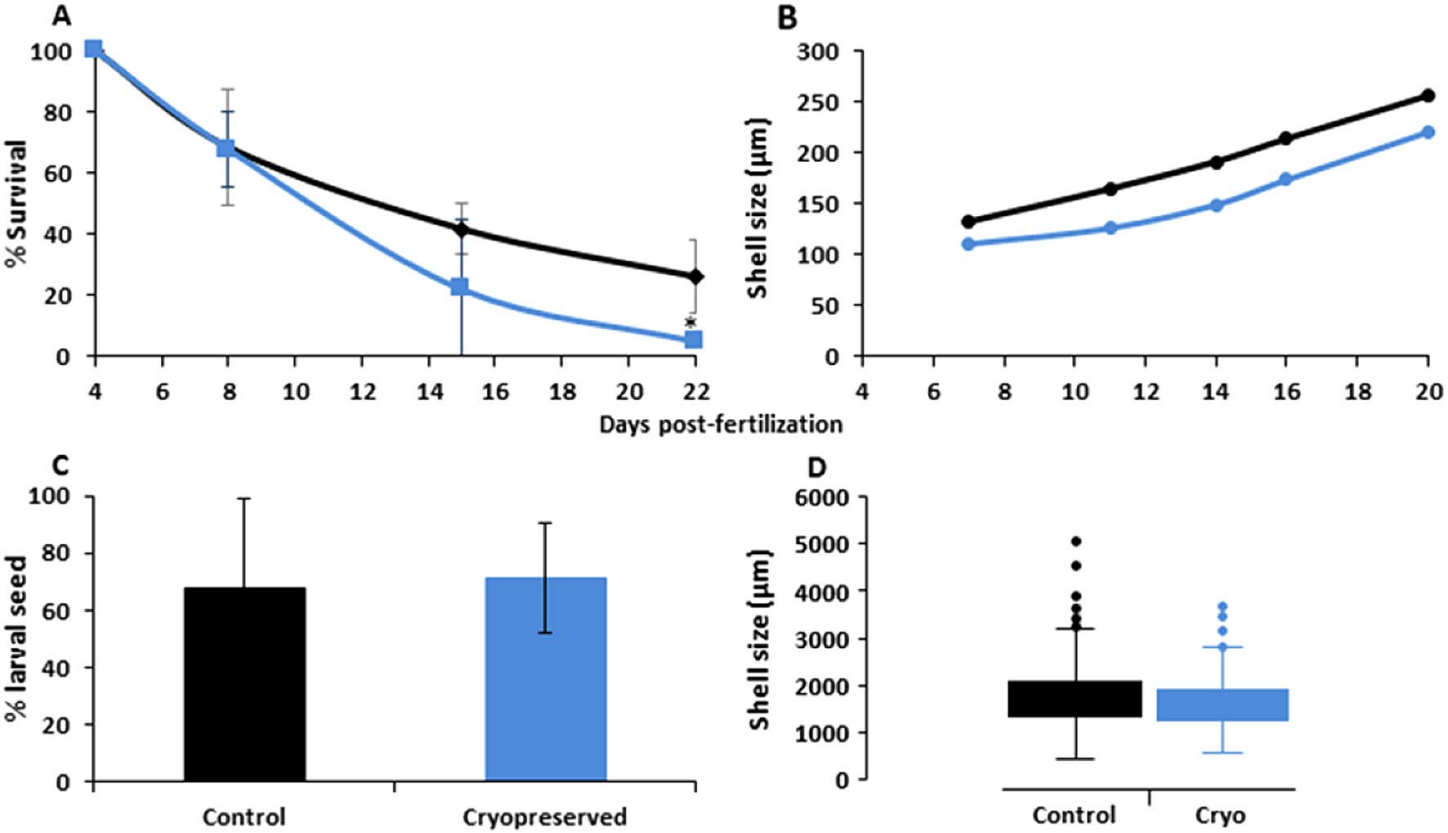
Fitness parameters (survival, larval shell size and settlement success) assessed over the long-term experiment carried out with F1 *Mytilus galloprovincialis* 72 h-old D-larvae. (**A**) Survival percentages of post-thawed larvae for 22 days, cultured in 150-L tanks (n = 4 tanks per treatment). Mean ± Standard Deviation (SD). (**B**) Shell growth of normal D-larvae throughout 22 days developed from cryopreserved (blue) or control (black) 72 h-old D-larvae (n = 35 per replicate). Mean ± Standard Deviation (SD). (**C**) Percentage of settled larvae developed from cryopreserved (blue) and control (black) larvae cultured in 50 cm^3^ settlement drums (150-µm mesh) (10,000 larvae/drum, n = 8 drums per treatment). Percentage settlement expressed as the proportion of juveniles obtained from the sub-sample of competent larvae studied in each treatment. Mean ± Standard Deviation (SD). (**D**) Box plots for the distribution of shell sizes in microns (µm) of settled larvae. The line inside the box represents the median value and box ends correspond to upper and lower quartiles. Upper and lower whiskers represent values outside the middle 50% quantile. Asterisk shows significant differences. p-value of ANOVA-one way test, significance was considered at p < 0.05.

Post-thaw larval parameters obtained during larval rearing and settlement are represented in Fig. 5. Regarding survival, there was a steep decline in larval density, especially between day 8 and 15 of culture, being more pronounced in cryopreserved samples. However, significant differences were not found between cryopreserved and control trials, excepting at day 22 (p < 0.05). The declining trend began to stabilize from day 16 onwards. At day 22, 5.24% of cryopreserved larvae developed into competent pediveliger larvae, representing 24% survival compared to the control group (Fig. 5A). Shell length was taken into account as an indicator of larval growth (Fig. 5B, Table 1). There was a significant difference in the size of of cryopreserved larvae on day 4 post-fertilization (control larvae: 116.47 ± 5.87 µm; cryopreserved larvae: 110.02 ± 5.51 µm, n = 35). This difference increased over the following 7 days of larval rearing until day 11, when shell length of cryopreserved larvae averaged 25% lower than that of controls (unfrozen larvae: 173.39 ± 22.30 µm; cryopreserved larvae: 129.71 ± 20.67 µm, n = 35). From day 12 onwards, these differences became less and no significant differences were found between larval size measurements at settlement (control larvae: 1775.03 ± 600.07 µm; cryopreserved larvae: 1600.99 ± 485.92 µm, n = 35) (p > 0.05).

**Table 1:** Larval size (μm), measured on normal D larvae and delay of cryopreserved larvae compared to control group.

Day post-fertilization	Shell size (µm)		Delay of cryopreserved larvae compared to control
	Control	Cryopreserved	
4	116.47 ± 5.87	110.02 ± 5.51	5.54
5	123.39 ± 6.35	109.78 ± 4.36	11.03
6	131.7 ± 8.00	111.26 ± 5.65	15.54
7	135.8 ± 12.04	112.99 ± 9.24	16.77
8	145.3 ± 13.02	118.37 ± 10.8	18.51
9	155 ± 15.97	120.95 ± 12.11	21.99
10	164.2 ± 17.99	126.77 ± 15.42	22.82
11	173.39 ± 22.29	129.71 ± 20.62	25.19
12	178 ± 23.04	138.07 ± 22.97	22.43
13	191.1 ± 29.4	149.63 ± 28.49	21.72
14	205.5 ± 32.06	158.45 ± 35.93	22.88
15	213.6 ± 34.99	173.87 ± 39.26	18.61
16	226.8 ± 35.62	190.12 ± 37.97	16.17
17	238.7 ± 34.56	191.97 ± 38.76	19.47
18	249.9 ± 35.13	214.20 ± 41.25	14.27
19	256.1 ± 32.56	220.24 ± 42.37	14.01
20	261.4 ± 40.04	232.73 ± 43.25	10.96
21	277.5 ± 36.09	251.16 ± 37.19	9.5
47	1583.25 ± 357.15	1432.19 ± 289.9	99.54
56	1755.03 ± 600.07	1600.99 ± 485.92	8.78

Mean ± Standard Deviation (SD), n = 35 per tank, 4 tanks per treatment.

Settlement analysis did not reveal significant differences when comparing successful percentage settlement values and shell sizes between treatments (p > 0.05). Settlement of mussel juveniles developed from cryopreserved larvae was 71.27 ± 19.10%, slightly higher than the 67.45 ± 31.65% of settled individuals (spat) from the control group (Fig. 5C). Focusing on the shell size of settled mussels, those from the control group reached a mean of 1755 ± 600.07 µm, whereas the cryopreserved group averaged 1595 ± 475.31 µm (Fig. 5D).

**Figure 6:**
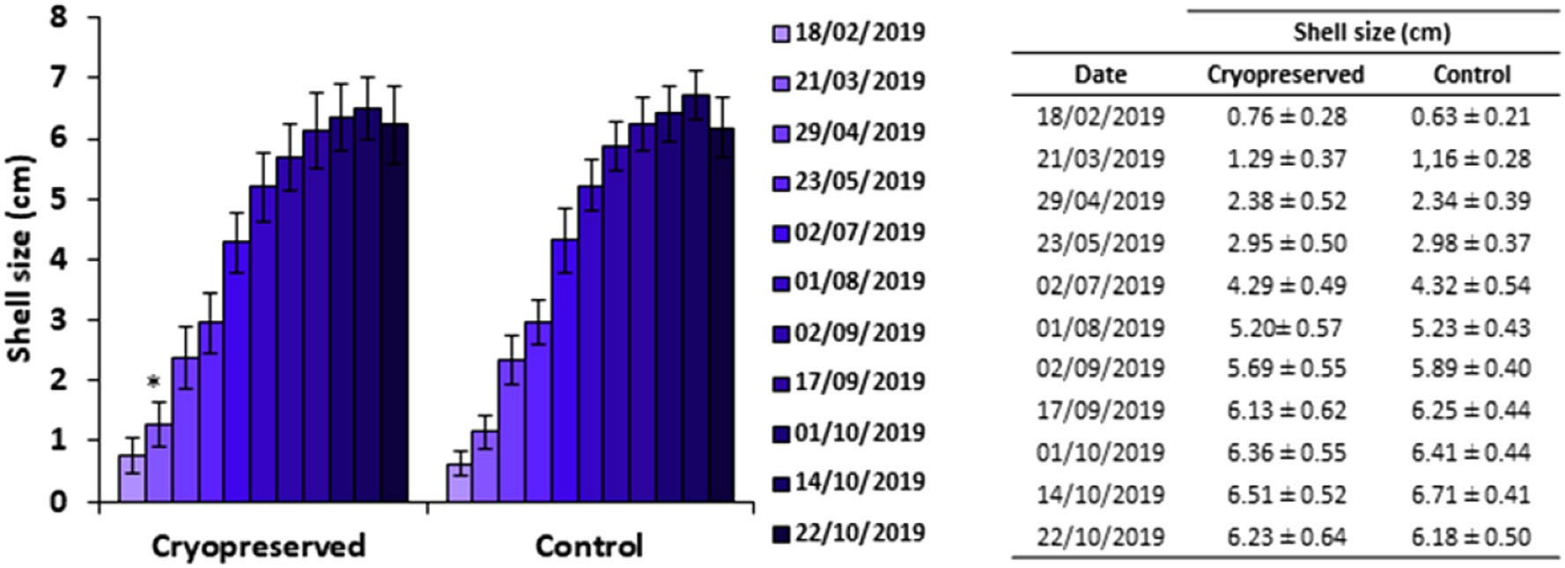
Shell size (cm) of mussels developed from cryopreserved larvae (F1) cultured in rafts in Ria de Vigo, NW Spain. Sampling dates shown as dd/mm/yyyy, results expressed as Mean ± Standard Deviation (SD) in centimeters (cm), n = 10 per culture rope, 4 ropes per treatment. Asterisk shows significant differences between treatments (p < 0.05).

In this experiment, the growth rate of developed adult mussels cultured in rafts in Ria de Vigo, (NW Spain) were evaluated (Fig. 6). In general, there were no significant differences between treatments and the growth trend seemed to follow a linear behavior in both cryopreserved and control groups until the final samplings, when shell size approached a maximum of about 6 cm.

#### Long-term experiment from cryopreserved F2 72 h-old D-larvae

**Figure 7:**
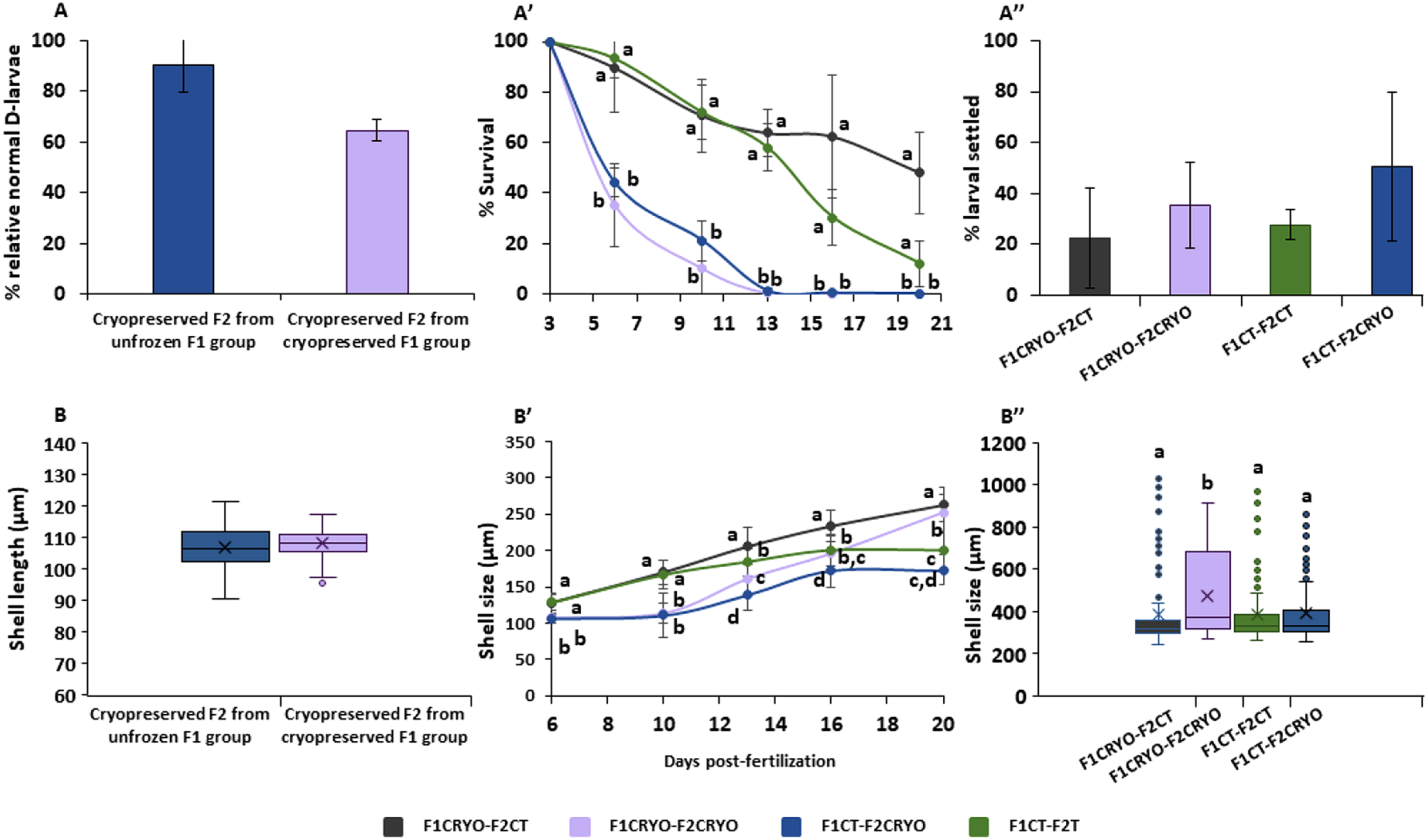
Fitness parameters (survival, larval shell size and settlement success) assessed over the long-term experiment carried out with F2 *Mytilus galloprovincialis* 72 h-old D-larvae. (**A**) Relative percentage of normal D-larvae obtained at day 2 of incubation after F2 72 h-old larval cryopreservation compared with control group. Mean ± Standard Deviation, SD (n = 100, 3 replicates per treatment). (**B**) Box plots for the distribution of shell sizes in microns (µm) of post-thawed larvae. The line inside the box represents the median value and box ends correspond to upper and lower quartiles. Upper and lower whiskers represent values outside the middle 50% quantile (n = 35, 3 replicates per treatment). (**Aʹ,Bʹ,Aʹʹ,Bʹʹ**) Post-thaw parameters of *Mytilus galloprovincialis* larvae after 72 h-old D-larval cryopreservation during 20 days of larval rearing. (**Aʹ**) Survival percentages (Mean ± Standard Deviation (SD), n = 3 tanks per treatment (150 L)). (**Bʹ**) Shell lengths (Mean ± Standard Deviation (SD), n = 3 tanks per treatment (150 L)) of developed larvae for 20 days. (**Aʹʹ**) Percentages of settled larvae per treatment (Mean ± Standard Deviation (SD), n = 3 tanks per treatment (50 cm^3^), initially, 1284 larvae per replicate). Percentage of settlement expressed as the proportion of juveniles obtained from the sub-sample of competent larvae studied in each treatment. (**Bʹʹ**) Box plots for the distribution of shell sizes in microns (µm) of settled juveniles. The line inside the box represents the median value and box ends correspond to upper and lower quartiles. Upper and lower whiskers represent values outside the middle 50% quantile (n = 35, 3 settlement drums per treatment (50 cm^3^)). *F1CRYO-F2CT* control larvae obtained from adults developed from cryopreserved larvae, *F1CRYO-F2CRYO* cryopreserved larvae obtained from adults developed from cryopreserved larvae, *F1CT-F2CT* control larvae obtained from adults developed from control larvae, *F1CT-F2CRYO* cryopreserved larvae obtained from adults developed from control larvae. Statistical significance was considered when p < 0.05.

The short-term experiment with F2 larvae did not show significant differences when comparing the relative percentage of normal D-larvae (Fig. 7A) and larval development (Fig. 7B) after cryopreservation. Cryopreservation of larvae from adults developed from control larvae yielded higher relative normality rates, 90 ± 10.91%, than cryopreserved F2 from F1 adults developed from cryopreserved larvae, with 64 ± 4.46%. However, significant differences between pools were not found (p > 0.05). Comparing shell lengths measured at day 2 of incubation, cryopreserved larvae from cryopreserved adults averaged 108 ± 4.46 µm (median 108.34 µm), whereas cryopreserved larvae from control adults achieved 106 ± 6.05 µm (median 106.52 µm), without statistical significance between treatments (p > 0.05).

During the first days of larval rearing of cryopreserved F2 larvae, the survival rate declined until day 13, after which the trend stablished until metamorphosis into pediveliger larvae at day 20. Statistical analysis showed significant differences between cryopreserved trials and control groups (p < 0.05). At the settlement point, the treatment F1CRYO-F2CONTROL produced 48% of normal pediveliger larvae (percentage expressed as the proportion of juveniles produced from the initial subsample of competent larvae after 10 days of incubation for each treatment), followed by F1CONTROL-F2CONTROL with 11.92%; whereas F1CRYO-F2CRYO yielded 0.15% and F1CONTROL-F2CRYO, 0.12% (Fig. 7Aʹ). Larval growth during the first 20 days of culture was also significantly different between F2 cryopreserved and control groups (p < 0.05), although, this parameter seemed to stabilize over time and the shell size of cryopreserved larvae tended to become more similar to the shell size of the control larvae (Fig. 7Bʹ). Regarding the settlement success, there was not any significant difference between treatments when comparing the percentage of settled larvae (p > 0.05). The treatment F1CONTROL-F2CRYO achieved the highest rate, with 50.54 ± 29.39%, followed by F1CRYO-F2CRYO, with 35.41 ± 16.70%. Around 25% of pediveliger larvae from control groups were able to settle (Fig. 7Aʹʹ). The lengths of settled juveniles (spat) were significantly different only between F1CRYO-F2CRYO (471 ± 202 µm) and the rest of groups (around 388 µm) (p < 0.05) (Fig. 7Bʹʹ).

### Discussion

New methodologies and good practices need to be developed for mollusk aquaculture to ensure a reliable supply of spat for aquaculture in order to meet increase demanded of the growing global population^3^. The implementation of breeding programs will be crucial for marine aquaculture, in the same way as it has already been successful for land agriculture. Selective breeding systems can be assisted by the application of cryopreservation tools, offering the possibility of storing interesting biological samples at high densities under minimal requirements compared to maintaining live stocks, able to survive and develop normally once they are thawed^20,22,38^. In addition to several advantages mentioned in the Introduction, the establishment of biobanks could be the way to overcome the logistical issues of obtaining and maintaining living shellfish broodstock, as well as lowering costs and effort^35^. For instance, shellfish hatchery requires daily a supply of diet composed of a mix of microalgal species in different proportions; continuous water flow and aeration are also needed, and the system must be efficient in terms of removing metabolites that can be deleterious. All of these activities can be time consuming, and expensive^39,40^.

This is the first time a cryopreservation protocol for *M. galloprovincialis* larvae has been investigated in depth from fertilization to juvenile and adult production, and production of a second generation. Our work started with a very basic preliminary protocol described for trochophore larva by Paredes et al.^25^, followed by studies on cryoprotectant toxicity^41^, cryopreservation of trochophore larvae and juvenile production^35^. A similar long-term experiment was carried out by Suquet et al.^1^, who evaluated the viability of progeny produced from cryopreserved oyster (*Crassostrea gigas*) larvae. However, the present research is pioneering in the field because we have studied the direct implementation of cryopreservation tools on aquaculture, focusing on the culture of the resulting spat in traditional mussel rafts in the natural environment and, in tandem, analysis of the potential long-term effects of cryopreservation on F2 progeny.

Improvements from our prior protocol for larvae were due to selection of the 72 h-old D-larval stage for cryopreservation, resulting not only in higher survival rates but also in increased quality of larval fitness. In addition, preference for the use of 72 h-old D-larvae is supported by our prior research that showed their higher tolerance to CPA toxicity and higher resistance to cryopreservation, compared with earlier development stages^35,41^. The optimized protocol produced close to 75% of normal D-larvae at day 2 of incubation, compared with only 25% success obtained with cryopreserved trochophores in a 48 h short-term post-thaw fitness evaluation^25,36^. Hence, by selecting older larval stages for cryopreservation, long-term outcomes could potentially be improved given the higher number of thawed normal D-larvae available for culture.

An explanation for the higher success when using an older stage larva, compared with trochophore larva, could be explained by Rusk^42^, who observed minor cell-specific damage and reduced neurogenesis in post-thawed trochophores. Other studies have explained larval mortality after thawing by higher oxidative stress from the production of reactive oxygen species (ROS) during the cryopreservation process^43–45^. Trochophores have higher lipid contents compared with older development stages. These molecules are susceptible to oxidative stress, which may lead to ROS production and significant irreversible cell damages. Although the potential metabolic pathways blocked by ROS have not been identified yet, research suggests that those involved on larval development could be affected, resulting in cell death^32,46,47^.

It is well known that selection of the optimal CPA and CPA concentration is species and cell specific. Hence, despite its detrimental effect on mussel development, dimethyl-sulfoxide (Me_2_SO) has been considered the preferred CPA for mussel sperm cryopreservation^41,48^, whereas research on mollusks has shown the success of using ethylene–glycol (EG) for larval cryopreservation^25,41,49^. In order to improve the cryopreservation success, the addition of non-permeable CPAs has been investigated, based on their capacity to avoid potential harmful effects of permeable CPAs and diminish osmotic shock^26,50–52^. The use of sugars, such as trehalose (TRE) is widely common, as well as polyvinylpyrrolidone (PVP)^25,35,36,49,53^.

Liu et al.^36^ recommended the use of PVP in combination with other CPAs to limit intracellular ice formation. However, the effect of PVP is unclear and they suggested further research focused on its toxicity to evaluate its potential harmful effect. The potential benefits of increasing PVP concentrations with 10% EG in combination or not with 0.4 M TRE were studied here, showing there was not any significant improvement.

On the other hand, the role of D-shell is unclear on CPA permeability. Further research on CPA permeability across tissues or complex cell aggregations and the role of barriers like the prodisoconch shell at the mussel D-stage could explain the differential responses of larval stages to cryopreservation. Moreover, 15 min of exposure seems to be enough time for osmotic equilibration to CPAs before slow cooling of mussel trochophores. However, research in our lab indicated^54^ the improvement of the post-thaw survival and larval fitness when increasing the exposure time from 15 to 60 min when cryopreserving 72 h-old D-larvae.

Several authors reported benefits from the use of an extender to help CPA removal from cells, which could help the cells reach osmotic equilibrium, diminish potential osmotic damage after thawing. Some sugars have been applied for these purposes previously, especially when cryopreserving the trochophore stage^25,36,49,53,55^. The final short-term experiment in this study tested the addition of increasing sucrose (SUC) concentrations in order to determine the optimal concentration to assist CPA removal. Despite the advantages reported in prior research, there was no enhancement of survival at any of the SUC tested concentrations.

This work has shown for the first time that cryopreserved mussel D-larvae can be reared to settlement and juveniles transferred to ropes and grown in the natural environment to produce a second generation. Long-term experiments suggested that cryopreservation does not compromise the development of larvae to adult stage. Adults that were cultured in traditional aquaculture systems showed similarities in growth rates; furthermore, histological analyses did not show any tissue alteration in cryopreserved individuals (data not shown). In addition, gametes of control and cryopreserved F1 had no differences in gamete quality, with oocytes with spherical shapes and high lipid droplet content and sperm with good motility and swimming in typical zig-zag trajectories. Moreover, larval rearing carried out with F2 larvae showed that cryopreservation of successive progenies did not affect production of mussel spat.

**Figure 8:**
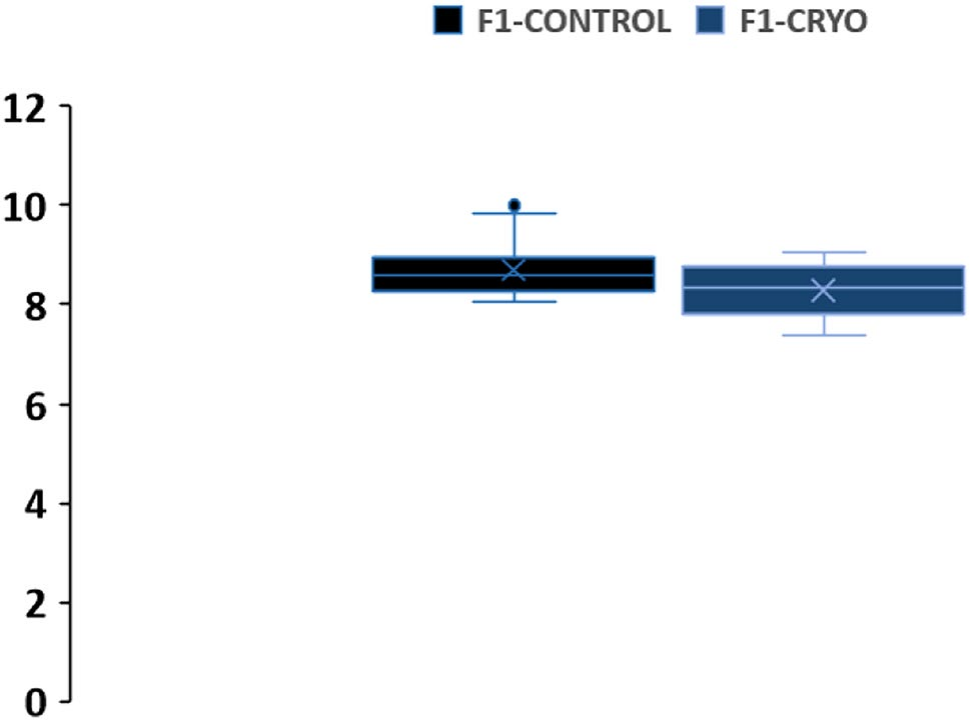
Box plots for the distribution of shell sizes in microns (µm) of F1 adult mussels cultured in traditional culture rafts in a natural environment, in Ria de Vigo (Spain). The line inside the box represents the median value and box ends correspond to upper and lower quartiles. Upper and lower whiskers represent values outside the middle 50% quantile (n = 10). Statistical signification was considered when p < 0.05.

Differences F1 and F2 larval survival rates could have been due to difficulties in the maintenance of larval cultures, given the unusual circumstances of the Covid-19 pandemic, which limited the opportunities to maintain optimal conditions for larval development. In addition, the age of developed adults (F1) could negatively influence gamete quality and undermine cryopreservation success: adults selected for spawning events to obtain the initial F1 D-larvae were 9 months to 1 year old, averaging 7 cm. On their part, resulting adults cultured in rafts (F1) were 2 years old at the time of spawning, measuring 8 ± 0.56 cm (those developed from control larvae) and 8 ± 0.51 cm (adults developed from cryopreserved larvae) (Fig. 8). Despite the unavoidable difference in F1 and F2 parental age, there was no significant difference between survival and settlement of cryopreserved F2 larvae from F1 parents that had been cryopreserved or not.

Differences in size were found between control and cryopreserved larvae mainly during the first 22 days of larval rearing. A similar effect was observed in Refs.^25,35,49,53^. Rusk et al.^53^ also observed a lower activity in cryopreserved samples compared to controls in other parameters, including feeding consumption and delays in organogenesis. These differences could be explained by considering potential cryo-injuries that may have occurred during cryopreservation on organs or tissues, for example effects on the mantle and shell gland which are responsible for shell secretions. This explanation is supported by the number of abnormalities that were found in the shells of cryopreserved larvae, as described by Rodríguez-Riveiro et al^35^ and Rusk et al.^53^. This might mean that cryopreserved larvae grow slower than controls, due to a re-allocation of energy from growth to processes involved in tissue repair. Those larvae with lethal injuries or serious damage could die during through larval rearing, depending on the degree of abnormality. At the settlement, larvae were competent and able to develop normally into juveniles and grow at the same rate as juveniles developed from control larvae. In fact, this is supported by the settlement success of F1 and F2 (Fig. 7Aʹʹ) offspring, where the number of settled juveniles (spat) from cryopreserved larvae was higher than controls (p > 0.05). Further research is needed to improve our understanding of the mechanisms underlying our results. Focusing on the development of mussel juveniles cultured in rafts in natural environment, shell sizes were similar among treatments. The normal size of adult mussels ranges from 3 to 13 cm in natural conditions and they can reach 7 cm at the end of the first year when the environment is favorable^56^. In this work, mussels reached 3 cm approximately 7 months after their transport to culture rafts and averaged 6–7 cm 1 year after. This observation indicates that cryopreservation does not have any detrimental effect on mussel growth, and they can reach commercial sizes as fast as individuals from non-cryopreserved larvae.

### Conclusions

The current work describes an optimized cryopreservation protocol for 72 h-old D larvae of *M. galloprovincialis* based on a suitable development stage, cooling and thawing rates and CPA combinations tested. For the first time, mussel spat produced from cryopreserved larvae were able to develop into adults at the same growth rates as control individuals, be cultured in a natural environment, and even reach average commercial size at the same time as control mussels obtained from non-cryopreserved larvae. In addition, the viability of these produced adults is apparently unaffected by the cryopreservation process, with fecundity and gamete quality equivalent to control mussels, which is corroborated by subsequent work on F2 mussels. Moreover, cryopreservation of larvae from the F2 generation does not compromise larval development. This represents robust evidence of the suitability of this cryopreservation method for aquaculture or research purposes where animals must possess optimal health. Further research should be focused on factors involved in the cryopreservation process, such as membrane permeability parameters, to improve post-thaw success to provide higher survival rates from the outset and diminish cell injury which can lead to long-term lethal damages. In addition, it will also be important to determine the processes involved in the high mortality rates found at the beginning of culture and the delay in development of cryopreserved larvae needs to be undertaken to understand affected intracellular processes and metabolic pathways, particularly during the first hours after thawing. This work shows the high potential of cryopreservation to benefit the mussel industry and the suitability of this technique for selective breeding programs, one of the most promising ways to increase global production.

## Materials and methods

### Gamete collection and handling

Mature blue mussels (*Mytilus galloprovincialis*, Lamark 1819) were obtained from the wild in the Ria de Vigo (Galicia, NW Spain) and deposited in PVC tanks supplied with filtered sea water (FSW; 35–37‰, filtered through 0.22-μm mesh and + UVA-treatment) at 18 ± 1 °C. Spawning of mussel adults was induced by thermal cycling according to Ref.^41^. Individuals that spawned freely were collected from the tray and put into 250-mL beakers separately. Oocyte quality and maturity were examined focusing on their shape and color before fertilization, selecting all oocyte pools with spherical shape, brownish-pinkish color and high content of lipid droplets. Sperm was checked for motility under the microscope and only samples with high zig-zag motion and no agglutination were retrieved. Two pools of sperm from two males and oocytes from three females were prepared for fertilization in order to minimize genetic variability^57,58^. A small volume of sperm was added to the oocyte suspension (approximately a ratio of 20:1, checked under the microscope) and a 15 min contact period was allowed before the evaluation of the fertilization percentage focusing on the formation of the polar body. The cells were incubated up to 72 h at 18 ± 1 °C in 150-L tanks. Trochophore larvae (20–24 h post-fertilization) and D-larvae of 72 h-old were retrieved for experiments.

### Reagents

Cryoprotecting agents (CPAs) were selected according to prior research on mussel cryopreservation^25,36,37,41,59^. All chemicals, ethylene–glycol (EG), trehalose (TRE), polyvinyl-pyrrolidone (PVP) and sucrose (SUC) were purchased from Sigma Aldrich Chemicals (St Louis, MO, USA). Cryoprotecting solutions were prepared at double the final concentration required in the experiment. Therefore, when the same volume of stock solution and larvae suspension were mixed, the final chemical concentration was as desired. CPA combinations were made up in FSW or Milli-Q water when needed.

### CPA addition and equilibration time for toxicity and cryopreservation trials

CPA combinations were added in one step at a 1:1 ratio to one mL of FSW with concentrated mussel larvae (400–600) at room temperature (18 ± 1 °C). Although there are no data for CPA permeation into mussel oocytes, embryos or larvae, 15 min were allowed for equilibration in the case of trochophores, considering empirical data that indicated that 15 min-exposure was time enough to reach osmotic equilibration^22,25,49,60^. For the D-larval stage, the exposure time was increased to 60 min, according to our prior research on mussel D-larval cryopreservation, where the increase in equilibrium time was beneficial in terms of post-thaw survival and larval fitness^37^. After exposure period, samples were incubated or cryopreserved following the guidelines explained below.

### Experimental design of short-term experiments

#### Potential toxic and cryoprotective effects of different CPA combinations with ethylene–glycol: trehalose (TRE) and polyvinylpyrrolidone (PVP)

Toxic and cryoprotection effects of different CPA solutions were studied on trochophore stage and 72 h-old D-larvae. From prior experiments, 10% (v/v) EG + 0.4 M (w/v) TRE was prepared either in FSW or Milli-Q water. Increasing concentrations of PVP (0.34, 1.7, 3, 9 and 12% w/v) were added to 10% (v/v) EG in FSW with the addition or not of 0.4 M TRE (final concentrations). CPAs were added to mussel individuals following the methodology described before, allowing equilibration. Larvae mixed with CPA solutions were loaded into 0.25-mL straws (IMV Technologies, France), then sealed with PVC powder. After equilibration, toxicity batches were diluted with FSW and prepared for incubation. In the case of samples destined for cryopreservation, straws were placed into a controlled rate freezer (Freeze Control System Cryologic Pty Ltd, Mt Waverley, Australia). The cryopreservation protocol was as described: holding at 4 °C for 2 min, then cooling at 1 °C/min to − 12 °C, holding for 2 min for seeding check, then cooling to − 35 °C with a rate of 1 °C/min, then plunging into liquid nitrogen (LN_2_) for storage. Thawing of straws took place by immersion in a 35 °C water bath for 6 s and incubated according to instructions described for short-term incubation conditions.

#### Effects of different cooling and thawing rates

Mussel trochophores and D-larvae were cryopreserved using two-step cooling rates: from 4 °C, samples were cooled at 1 °C/min to − 12 °C (seeding), then three different cooling rates were tested: 1 °C/min, 0.5 °C/min or 0.3 °C/min to − 35 °C, then plunging into LN_2_. Straws were thawed using four water baths at increasing temperatures (at 18, 28, 35 and 40 °C) until the ice visibly melted. CPA solutions containing 10% EG + 0.4 M TRE were prepared in either FSW for D-larval stage or in Milli-Q Water for trochophore larvae, based on prior toxicity and cryopreservation outcomes.

#### Effects of SUC solutions on post-thawed D-larvae at the CPA dilution step

For this experiment, the cryopreservation protocol was chosen according to the best outcome obtained in the previous cryopreservation test, which consisted of holding at 4 °C for 2 min, then cooling at − 1 °C/min to − 12 °C, holding for 2 min for seeding, then cooling at − 1 °C/min to − 35 °C, then plunging into LN_2_. Thawing was performed using a 35 °C water bath. In this case, the content of thawed straws was diluted with SUC solutions (6, 9, 12 and 15%) prepared in FSW or Milli-Q water in one step at a 1:1 ratio by volume. After 2 min of equilibration at room temperature (18 ± 1 °C), samples were filtered and rinsed in 20 mL FSW. Then, samples were incubated for 24 h or 48 h at 18 ± 1 °C.

#### Short-term incubation conditions

Samples were diluted and rinsed with FSW 1:1 using a 40-μm membrane filter to remove the CPAs. Then, they were incubated in 20 mL of clean FSW at 18 ± 1 °C (incubation density of 20–40 larvae/mL) for 24 h in the case of trochophores for assessment of developmental success into D-larval stage. In the case of D-larvae, incubation was for 48 h. At the end of incubation, cells were fixed with formalin. The control group was a sub-sample of fresh larvae incubated in 20 mL FSW. Three replicates were assayed for each CPA solution and control treatments. Further assessment of CPA toxicity and cryopreservation outcome are explained in detail the section (below) on Larval abnormality criteria.

### Long-term experiments

#### Long-term experiment from cryopreserved F1 72 h-old D-larvae

Fertilized eggs obtained from in-vitro fertilization after spawning male and female adults were incubated in two 150-L tanks at 18 ± 1 °C and a concentration of 0.03 × 106 cells/L. Subsequently, D-larvae at 72 h-old were collected by siphoning the contents of the tanks on a 40-μm screen that was semi-submerged to avoid mechanical damage. D-larvae were concentrated in 3 mL FSW for cryopreservation. The CPA solution and cryopreservation protocol were selected according to the best results from previous work: the combination of 10% EG + 0.4 M TRE in FSW was added to 1 mL of FSW with D-larvae at 1:1 ratio (by volume) in one step. After 60 min of equilibration, 0.25-mL straws were filled with the larval suspension (307,371–496,000 larvae/straw, 15–21 straws per experiment), sealed with PVC powder and introduced to the controlled rate freezer. The cryopreservation protocol started by holding at 4 °C for 2 min, then cooling at 1 °C/min to − 12 °C, holding for 2 min for a seeding check, then cooling to − 35 °C at 1 °C/min, then plunging into LN2 for storage. Thawing of the straws took place by immersion in a 35 °C water bath for 6 s. Initially, the number of cryopreserved D-larvae was approximately one-third higher than the number of larvae destined for control trials. This was designed taking into account our previous short-term cryopreservation experiments, which showed that the optimized cryopreservation protocol produced around 75% of normal D-larvae after 48 h post-thawing. Therefore, after 48 h cryopreservation both cryopreserved and control trials would be expected to have the same density at the beginning of the larval rearing.

Larval culture was from thawing to settlement. Larvae were cultured in 150-L tanks (n = 4 per treatment, initial density in control tanks (day 1): 5.45 larvae/mL; initial density in cryopreserved trials: 6.61 larvae/mL). They were maintained at 18 ± 1 °C with constant aeration provided by glass tubes. Feeding consisted of 60 to 100 equivalents to *Isochriysis galbana* (mix of *Tisochrysis lutea*, *Rhodomonas lens*, *Chaetoceros neogracile*, *Phaeodactylum tricornutum* and *Tetraselmis suecica*), as described in Ref.^61^. Larval samples were taken twice a week to obtain survival rates and larval sizes (n = 35 for each tank) during larval rearing. From day 22 onwards, mussel juveniles were transferred to settlement drums (150-μm mesh) and incubated for 10 days to enable the settlement. On the last day of culture, settled mussels were collected for a final counting and a subsample was further cultured in PVC tanks (50 m^3^) in FSW at 18 ± 1 °C with culture ropes to allow their settlement on the rope. After 27 days under these conditions, the ropes were transported to culture rafts in the Ria of Vigo for monitoring of mussel growth and development in natural conditions. However, at the beginning of this period in the ocean, ropes were covered by a mesh to avoid potential predation and settlement of wild seed. Once mussels were around 2–3 cm, the protective mesh was removed and size measurements were taken periodically during the following months (n = 10 per rope, 4 ropes per treatment). Mussels and ropes were scraped and cleaned, eliminating associated fauna as well as smaller wild seed from subsequent seasonal spawning of natural mussel populations.

#### Long-term experiment from cryopreserved F2 72 h-old D-larvae

Cultured F1 adult mussels were supposed to be collected after 1 year growing in their natural environment, but finally this period in the ocean lasted 2 years due to the COVID-19 pandemic and the impossibility to proceed with the experiment given lockdown restrictions in Spain. When conditions were favorable, they were transported to the lab close to their natural spawning season and incubated in PVC trays in an open-water system and fed to optimize gonad maturation. Spawning inductions were carried out to ensure that the gametes from mussels developed from control larvae remained separated from the adults developed from cryopreserved larvae. After fertilization, fertilized eggs of both treatments were cultured separately in 150-L tanks (0.04 × 106/L) at 18 ± 1 °C. After 72 h, D-larvae were collected and concentrated, following the methods described in the previous experiment. A subsample of each pool was retrieved for a short-term cryopreservation experiment. For the long-term experiment, approximately two-thirds of both larval pools were cryopreserved methods described above (2.5.1 section), as well as for larval rearing during the first 20 days starting from thawing. Larvae were incubated in 150-L tanks (n = 3 per treatment, initial density in control tanks (day 1): 5.14 larvae/mL; initial density in cryopreserved trials: 7.17 larvae/mL) at 18 ± 1 °C with constant aeration and fed as described above. Periodical sampling was carried out twice a week through the larval rearing to obtain survival rate and larval size (n = 35 for each tank) parameters. At day 20, larvae developed an eye spot and foot, therefore a sub-sample of each treatment was transferred to settlement drums (hollow PVC cylinder provided by 150-μm mesh in one of the bases, which is submerged in FSW) for culture of 10 days. At the end of this settlement period, juvenile mussels were collected and fixed to assess settlement success (expressed as percentage of juveniles settled based on the size of the number of competent larvae).

### Larval abnormality criteria

Percentage of abnormal D-larvae was calculated as an indicator of the toxicity of the CPAs. On the contrary, the percentage of normal D-larvae developed after thawing was used to assess cryopreservation outcome. Both parameters were obtained after examining n = 100 cells for each replicate and treatment, under the microscope Nikon ECLIPSE 2000-5 and using Nikon NIS Elements-D software, version 4.13. The discrimination between normal and abnormal D-larvae was determined under a microscope attending to previous work focused on shell larval morphology and guidelines from other experts in the shell abnormalities and abnormally developing larvae of related mollusks in ecotoxicological larval bioassays^25,53,54,62,63^. Typical larval abnormalities found included delayed development, deviations from the D-larvae shell shape like indented margins or hinge deformations (concave or convex hinges) or presence of clear protruding mantle.

### Statistical analysis

Statistical analyses were performed according to Newman^64^ and Sokal and Rohlf^65^ and using the software SPSS v15.0. Normality of data distribution was tested using the Shapiro–Wilk test or Kolmogorov–Smirnov test when corresponding (p > 0.05) while homogeneity of variances was checked using the Levene’s test (p > 0.05). All percentage data were arcsine square-root transformed to improve normality^66^. When normality and homogeneity of variances were achieved, data were analyzed by one-way analysis of variance (ANOVA) followed by Dunnett or Bonferroni’s post-hoc tests. For analysis of size measurements, pair-wise comparisons between treatments were performed using non-parametric Mann–Whitney U test. Significant difference was accepted at p < 0.05.

### Significance statement

This work contains in-depth studies on crucial factors for the improvement of the cryopreservation protocol for mollusk larval stages. Moreover, potential cryopreservation effects were investigated long-term in F1 and F2 cryopreserved larvae. For the first time, the capacity of cryopreserved larvae completely able to develop into adult Mediterranean mussels (*Mytilus galloprovincialis*) cultured in natural environment was showcased. They can produce a second generation, which can develop normally, and their cryopreservation can produce competent mussel spat. These findings contribute positively to the field by providing robust evidence that cryopreservation techniques could be implemented in selective breeding programmes for aquaculture and research purposes given its capacity to produce individuals in optimal conditions.

## Data Availability

The datasets generated and/or analysed during the current study are available in the Integrated Marine Information System (IMIS) repository, 10.14284/526.

## References

[1] Suquet M (2014). Survival, growth and reproduction of cryopreserved larvae from a marine invertebrate, the Pacific Oyster (*Crassostrea gigas*). PLoS ONE.

[2] Widdows J (1991). Physiological ecology of mussel larvae. Aquaculture.

[3] Asociación Empresarial de Acuicultura en España (APROMAR) (2020). Aquaculture in Spain 2020.

[4] FAO. *Mytilus galloprovincialis, Food and Agriculture Organization of the United Nations (FAO)* (Fisheries and Aquaculture Department). http://www.fao.org/fishery/culturedspecies/Mytilus_galloprovincialis/en-consulted (Accessed 06 September 2021).

[5] Goulletquer P, Héral M (1997). Marine molluscan production trends in France: From fisheries to aquaculture. NOAA Tech. Rep. NMFS.

[6] Goulletquer P, Le Moine O (2002). Shellfish farming and coastal zone management (CZM) development in the Marennes-Oléron Bay and Charentais Sounds (Charente Maritime, France): A review of recent developments. Aquacult. Int..

[7] Béchemin C (2015). Episodes de mortalité massive de moules bleues observés en 2014 dans les Pertuis Charentais Bull. Epidémiol. Hebd..

[8] Polsenaere P (2017). Potential environmental drivers of a regional blue Mussel mass mortality event (winter of 2014, Breton Sound, France). J. Sea Res..

[9] Benabdelmouna A (2018). Mortality investigation of *Mytilus edulis* and *Mytilus galloprovincialis* in France: An experimental survey under laboratory conditions. Aquaculture.

[10] Charles M (2020). High mortality of mussels in northern Brittany—Evaluation of the involvement of pathogens, pathological conditions and pollutants. J. Invertebr. Pathol..

[11] Dickie LM (1955). Fluctuation in abundance of the giant scallop, *Placopecten magellanicus* (Gmelin) in the Digby area of the Bay of Fundy. J. Fish. Res. Board Can..

[12] Paulet YM, Bekhadra F, Devauchelle N, Donval A, Dorange G (1997). Cycles saisonniers, reproduction et qualité des ovocytes chez *Pecten maximus* en rade de Brest. Ann. l’Inst. Océanogr..

[13] Avendaño M, Cantillánez M, Riascos JM (2019). The decreasing availability of settlement surfaces affects the transition from larvae to early recruitment of the scallop *Argopecten purpuratus* through El Niño and La Niña episodes. Front. Mar. Sci..

[14] Díaz S (2016). Long-term epidemiological study of disseminated neoplasia of cockles in Galicia (NW Spain): Temporal patterns at individual and population levels, influence of environmental and cockle-based factors and lethality. J. Fish Dis..

[15] Beiras R (2003). Integrative assessment of marine pollution in Galician estuaries using sediment chemistry, mussel bioaccumulation, and embryo-larval toxicity bioassays. Chemosphere.

[16] Yancheva V (2018). Mussels in ecotoxicological studies—Are they better indicators for water pollution than fish?. Ecol. Balkanica.

[17] Azizi G (2020). Assessment of Heavy Metals (Fe, Cu and Ni) Contamination of Seawater and Mussel, Mytilus galloprovincialis, from Al Hoceima Moroccan Coasts.

[18] Boudry P, Dégremont L, Haffray P, Samain JF, McCombie H (2008). The genetic basis of summer mortality in Pacific oyster spat and potential for improving survival by selective breeding. France in Summer mortality of Pacific oyster—The Morest Project.

[19] Piferrer F (2009). The use of induced polyploidy in the aquaculture of fish and shellfish for performance improvement and genetic containment. Aquaculture.

[20] Symonds JE (2019). Developing successful breeding programs for New Zealand aquaculture: A perspective on progress and future genomic opportunities. Front. Genet..

[21] Hall SA, Méthé D, Stewart-Clark SE, Fraser K, Clark R (2020). Tremblay, comparison of absorption efficiency and metabolic rate between wild and aquaculture oysters (*Crassostrea virginica*). Aquacult. Rep..

[22] Adams SL (2009). Towards cryopreservation of Greenshell™ mussel (*Perna canaliculus*) oocytes. Cryobiology.

[23] Paredes E (2016). Biobanking of a marine invertebrate model organism: The Sea Urchin. J. Mar. Sci. Eng..

[24] Zhang TT, Fuller BJ, Lane N, Benson EE (2004). Cryopreservation of gametes and embryos of aquatic species. Life in the Frozen State.

[25] Paredes E, Bellas J, Adams SL (2013). Comparative cryopreservation study of trochophore larvae from two species of bivalves: Pacific oyster (*Crassostrea gigas*) and Blue mussel (*Mytilus galloprovincialis*). Cryobiology.

[26] Paredes E (2015). Exploring the evolution of marine invertebrate cryopreservation–Landmarks, state of the art and future lines of research. Cryobiology.

[27] Labbé C (2018). Cryopreservation of Pacific oyster (*Crassostrea gigas*) larvae: Revisiting the practical limitations and scaling up the procedure for application to hatchery. Aquaculture.

[28] Di Matteo O, Langellotti AL, Masullo P, Sansone G (2009). Cryopreservation of the Mediterranean mussel (*Mytilus galloprovincialis*) spermatozoa. Cryobiology.

[29] Smith JF (2012). Cryopreservation of Greenshell™ mussel (*Perna canaliculus*) sperm, I. Establishment of freezing protocol. Aquaculture.

[30] Smith JF (2012). Cryopreservation of Greenshell™ mussel (*Perna canaliculus*) sperm. II. Effect of cryopreservation on fertility, motility, viability and chromatin integrity. Aquaculture.

[31] Tervit HR (2005). Successful cryopreservation of Pacific oyster (*Crassostrea gigas*) oocytes. Cryobiology.

[32] Gale SL, Burritt DJ, Tervit HR, Adams SL, McGowan LT (2014). An investigation of oxidative stress and antioxidant biomarkers during Greenshell mussel (*Perna canaliculus*) oocyte cryopreservation. Theriogenology.

[33] Campos S, Troncoso J, Paredes E (2021). Major challenges in cryopreservation of sea urchin eggs. Cryobiology.

[34] Paniagua-Chavez CG, Buchanan JT, Supan JE, Tiersch TR (1998). Settlement and growth of Eastern oysters produced from cryopreserved larvae. Cryo-Letters.

[35] Rodríguez-Riveiro R, Heres P, Troncoso J, Paredes E (2019). Long term survival of cryopreserved mussel larvae (*Mytilus galloprovinciallis*). Aquaculture.

[36] Liu Y (2020). Development of a programmable freezing technique on larval cryopreservation in *Mytilus galloprovincialis*. Aquaculture.

[37] Paredes E, Heres P, Anjos C, Cabrita E, Wolkers W, Oldenhof H (2021). Cryopreservation of marine invertebrates: From sperm to complex larval stages. Cryopreservation and Freeze-Drying Protocol, Methods in Molecular Biology.

[38] Macavoy ES, Wood AR, Gardner JPA (2008). Development and evaluation of microsatellite markers for identification of individual Grenshell™ mussels (*Perna canaliculus*) in a selective breeding programme. Aquaculture.

[39] Pronker E, Nevejan NM, Peene F, Geijsen P, Sorgeloos P (2008). Hatchery broodstock conditioning of the blue mussel *Mytilus edulis* (Linnaeus 1758). Part I. Impact of different micro-algae mixtures on broodstock performance. Aquacult. Int..

[40] Ragg NLC, King N, Watts E, Morrish J (2010). Optimizing the delivery of the key dietary diatom *Chaetoceros calcitrans* to intensively cultured Greenshell™ mussel larvae, *Perna canaliculus*. Aquaculture.

[41] Heres P, Rodríguez-Riveiro R, Troncoso J, Paredes E (2019). Toxicity tests of cryoprotecting agents for *Mytilus galloprovincialis* (Lamark, 1819) early developmental stages. Criobiology.

[42] Rusk B (2012). Larval Development of the New Zealand Mussel Perna canaliculus and Effects of Cryopreservation.

[43] Len JS, Koh W, Tan SX (2019). The roles of reactive oxygen species and antioxidants in cryopreservation. Biosci. Rep..

[44] Gualtieri R (2021). Mitochondrial dysfunction and oxidative stress caused by cryopreservation in reproductive cells. Antioxidants.

[45] Castro PL, Ferraz A, Patil JG, Ribeiro RP (2021). Use of melatonin as an inhibitor of apoptotic process for cryopreservation of zebrafish (*Danio rerio*) embryos. Braz. J. Biol..

[46] Takahashi S, Ando A, Takagi H, Shima J (2009). Insufficiency of copper ion homeostasis causes freeze-thaw injury of yeast cells as revealed by indirect gene expression analysis. Appl. Environ. Microbiol..

[47] Coffman JA, Coluccio A, Planchart A, Robertson AJ (2009). Oral–aboral axis specification in the sea urchin embryo: III. Role of mitochondrial redox signaling via H2O2. Dev. Biol..

[48] Liu Y, Li X, Robinson N, Qin J (2015). Sperm cryopreservation in marine mollusk: A review. Aquacult. Int..

[49] Paredes E (2012). Cryopreservation of Greenshell™ mussel (*Perna canaliculus*) trochophore larvae. Cryobiology.

[50] Adams SL (2008). Application of sperm cryopreservation in selective breeding of the Pacific oyster, *Crassostrea gigas* (Thunberg). Aquac. Res..

[51] Hassan Md, Qin JG, Li X (2015). Sperm cryopreservation in oysters: A review of its current status and potential for future in aquaculture. Aquaculture.

[52] Liu B (2015). Cryopreservation of strip spawned sperm using non-programmable freezing technique in the blue mussel *Mytilus galloprovincialis*. Aquacult. Res..

[53] Rusk AB, Alfaro AC, Young T, Watts E, Adams SL (2020). Development stage of cryopreserved mussel (*Perna canaliculus*) larvae influences post-thaw impact on shell formation, organogenesis, neurogenesis, feeding ability and survival. Cryobiology.

[54] Heres P, Troncoso J, Paredes E (2021). Larval cryopreservation as new management tool for threatened clam fisheries. Sci. Rep..

[55] Liu Y, Li X (2015). Successful oocyte cryopreservation in the blue mussel *Mytilus galloprovincialis*. Aquaculture.

[56] Picker MD, Griffiths CL (2011). Alien and Invasive Animals—A South African Perspective.

[57] Stebbing, W.H. *et al*. *The role of bioassays in marine pollution monitoring in Bioassay Panel Report, Rapports et Process-verbaux des Reunions du Conseil Permanent International pour I'Exploration de la Mer*, Vol. 179, 322–332 (1980).

[58] Klöckner K, Rosenthal H, Willführ J (1985). Invertebrate bioassays with North Sea water samples. I. Structural effects on embryos and larvae of serpulids, oysters and sea urchins. Helgoländer Meeresunters.

[59] Heres P (2020). Development of a method to cryopreserve Greenshell mussel™ (*Perna canaliculus*) veliger larvae. Cryobiology.

[60] Adams SL (2004). Cryopreservation of sperm of the Pacific oyster (*Crassostrea gigas*): Development of a practical method for commercial spat production. Aquac. Res..

[61] Muller-Feuga A, Robert R, Calu C, Robin J, Divanach P, Stǿttrup JG, McEvoy LA (2003). Uses of microalgae in aquaculture. Live Feeds in Marine Aquaculture.

[62] His E, Seaman MN, Beiras R (1997). A simplification the bivalve embryogenesis and larval development bioassay method for water quality assessment. Water Res..

[63] Ventura A, Sculz S, Dupont S (2016). Maintained larval growth in mussel larvae exposed to acidified under-saturated seawater. Sci. Rep..

[64] Newman MC (1995). Quantitative Methods in Aquatic Ecotoxicology. Advances in Trace Substances Research.

[65] Freemand WH (1995). Biometry: The Principles and Practice of Statistics in Biological Research.

[66] Hayes WJ, Hayes WJ, Laws ER (1991). Dosage and other factors influencing toxicity. Handbook of Pesticide Toxicology.

